# A spectroscopic liquid biopsy for the earlier detection of multiple cancer types

**DOI:** 10.1038/s41416-023-02423-7

**Published:** 2023-09-16

**Authors:** James M. Cameron, Alexandra Sala, Georgios Antoniou, Paul M. Brennan, Holly J. Butler, Justin J. A. Conn, Siobhan Connal, Tom Curran, Mark G. Hegarty, Rose G. McHardy, Daniel Orringer, David S. Palmer, Benjamin R. Smith, Matthew J. Baker

**Affiliations:** 1 Dxcover Ltd., Royal College Building, 204 George Street, Glasgow, G1 1XW UK; 2 Translational Neurosurgery, Centre for Clinical Brain Sciences, University of Edinburgh, Edinburgh, EH4 2XU UK; 3 Department of Pure and Applied Chemistry, University of Strathclyde, Thomas Graham Building, 295 Cathedral Street, Glasgow, G11XL UK; 4 Children’s Mercy Research Institute, Children’s Mercy Kansas City, 2401 Gillham Rd, Kansas City, 64108 MO USA; 5 Department of Neurosurgery, New York University Grossman School of Medicine, New York, NY 10018 USA; 6 School of Medicine, Faculty of Clinical and Biomedical Sciences, University of Central Lancashire, Preston, PR1 2HE UK

**Keywords:** Cancer, Cancer, Diagnostic markers, Preclinical research

## Abstract

**Background:**

A rapid, low-cost blood test that can be applied to reliably detect multiple different cancer types would be transformational.

**Methods:**

In this large-scale discovery study (*n* = 2092 patients) we applied the Dxcover® Cancer Liquid Biopsy to examine eight different cancers. The test uses Fourier transform infrared (FTIR) spectroscopy and machine-learning algorithms to detect cancer.

**Results:**

Area under the receiver operating characteristic curve (ROC) values were calculated for eight cancer types versus symptomatic non-cancer controls: brain (0.90), breast (0.76), colorectal (0.91), kidney (0.91), lung (0.91), ovarian (0.86), pancreatic (0.84) and prostate (0.86). We assessed the test performance when all eight cancer types were pooled to classify ‘any cancer’ against non-cancer patients. The cancer versus asymptomatic non-cancer classification detected 64% of Stage I cancers when specificity was 99% (overall sensitivity 57%). When tuned for higher sensitivity, this model identified 99% of Stage I cancers (with specificity 59%).

**Conclusions:**

This spectroscopic blood test can effectively detect early-stage disease and can be fine-tuned to maximise either sensitivity or specificity depending on the requirements from different healthcare systems and cancer diagnostic pathways. This low-cost strategy could facilitate the requisite earlier diagnosis, when cancer treatment can be more effective, or less toxic.

**Statement of translational relevance:**

The earlier diagnosis of cancer is of paramount importance to improve patient survival. Current liquid biopsies are mainly focused on single tumour-derived biomarkers, which limits test sensitivity, especially for early-stage cancers that do not shed enough genetic material. This pan-omic liquid biopsy analyses the full complement of tumour and immune-derived markers present within blood derivatives and could facilitate the earlier detection of multiple cancer types. There is a low barrier to integrating this blood test into existing diagnostic pathways since the technology is rapid, simple to use, only minute sample volumes are required, and sample preparation is minimal. In addition, the spectroscopic liquid biopsy described in this study has the potential to be combined with other orthogonal tests, such as cell-free DNA, which could provide an efficient route to diagnosis. Cancer treatment can be more effective when given earlier, and this low-cost strategy has the potential to improve patient prognosis.

## Introduction

Earlier detection of cancers is of vital importance in improving patient survival, since earlier diagnosis and treatment can maximise the opportunity to combat or control disease progression. When cancer is diagnosed at an earlier stage, surgical resection is more often achievable, reducing or avoiding the toxicity of radiotherapy or chemotherapy—a reported 70% of early-stage tumours (Stage I) are treated with surgery, whereas only ~13% of Stage IV cancers undergo resection [1]. Delayed diagnosis can permit local cancer spread and metastasis, with a commensurate poorer prognosis as therapies are less effective for late-stage disease [2]. Most cancers are not screened for, often because of the low prevalence in the general population and corresponding high cost per cancer detected [3]. Employing a rapid liquid biopsy platform that can support clinicians in the diagnosis of different cancers would be transformational, particularly for patients who develop cancers not targeted in screening programmes. This strategy would also be useful for patients with non-specific symptoms, where the site of a potential cancer is uncertain. In fact, the early symptoms of many cancers are non-specific, and their significance can be easily overlooked by both patients and doctors. When symptoms are not readily indicative of a single organ to investigate, the ordering of tests can be delayed. A test that is low-cost, rapid, and capable of being applied to detecting different cancer types would therefore have a lower barrier for use. Application of the test could help clinicians ‘rule-out’ patients where the clinical suspicion of cancer was low, and provide an enriched cohort of ‘at risk’ patients to be prioritised for rapid diagnostic investigation according to standard care pathways for suspected cancer.

**Fig. 1:**
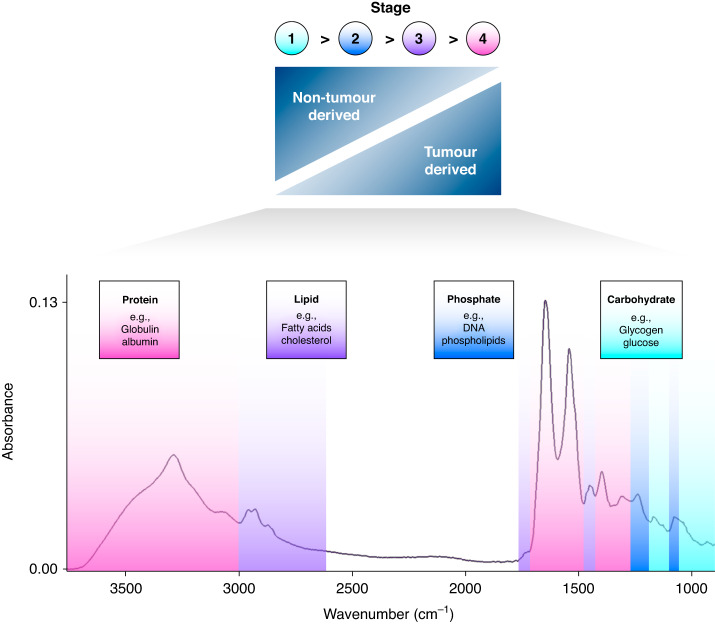
A typical biological spectrum with highlighted biomolecular peak assignments. The technique is sensitive to both tumour and non-tumour-derived information, making it applicable for early-stage cancer detection.

Liquid biopsies are under development that can identify a wide range of molecular features that may be indicative of cancer, derived either from the tumour itself or from the body’s response to the tumour [4]. Multi-cancer tests have recently been assessed for screening [5, 6], as well as for symptomatic patients referred for cancer investigation [7]. Many of these technologies are based upon genomic methods utilising the identification of genetic material, such as circulating tumour DNA (ctDNA) and/or cell-free DNA (cfDNA) [8]. However, there are significant limitations when exclusively detecting these markers. The release of ct/cfDNA into the bloodstream is highly variable, and not all cancer types and sub-types of a particular cancer shed enough material for reliable detection [9]. Early-stage cancers shed such low amounts of ctDNA that it is often beyond the detection capability of the current techniques [5], such that it has been likened to searching for a ‘needle in a haystack’ [10]. In addition, the cost of genetic-based methodologies remains high, limiting the likelihood that such a strategy can be effectively implemented at a population level for patients with non-specific symptoms where the incidence of cancer is low. While tumour-derived signals are usually more abundant in late-stage cancer, the signals for non-tumour-derived sources—e.g., immune response—dominate in early-stage disease. A pan-omics approach combining both tumour- and non-tumour-derived signatures could address the intrinsic limitations of genetic-based liquid biopsies [11]. A promising alternative strategy for earlier cancer detection is the clinical use of Fourier transform infrared (FTIR) spectroscopy. This method—which has been extensively described and evaluated in previous publications [12–14]—probes a wide range of biological features, producing a distinctive signature that represents the whole biomolecular profile of the sample and is inclusive of the full range of diagnostic information from both the tumour and the non-tumour response (Fig. 1) [15].

Attenuated total reflection (ATR-)FTIR spectroscopy is particularly well-suited for the clinic as the methodology is low-cost, requires little to no sample preparation, and provides reproducible results in a matter of minutes [16]. A previous study using ATR-FTIR spectroscopy showed that the generated signature can be used to predict brain cancer with a 96% sensitivity when combined with machine-learning techniques [17]. The spectroscopic liquid biopsy approach can be fine-tuned to maximise either sensitivity or specificity depending on the requirements of specific international healthcare systems and the diagnostic pathways for certain cancers. In this large-scale discovery-stage study, covering approximately the top 50% of the global cancer burden [18], the Dxcover® Cancer Liquid Biopsy has been assessed upon its ability to predict individual cancers in organ-specific classifications (brain, breast, colorectal, kidney, lung, ovarian, pancreatic and prostate). We also made a further exploratory evaluation of the ability to differentiate the signature from any one of the eight cancers from non-cancer patient samples.

## Methods

### Patient samples

All samples for patients eligible for inclusion in this study were sourced from biobanks (see Supplementary Text for more information). All cancer samples were collected from patients with a histopathological confirmed cancer diagnosis according to the data collection methods of specified biobanks. Samples were collected before surgical resection or the start of other anti-cancer therapies. The non-cancer group was comprised of both asymptomatic controls and patients with suspicious symptomology. The full cohort consisted of 2092 patients, of which 1542 had a confirmed cancer diagnosis (Supplementary Table S1). The (C) cancer set includes patients with either brain, breast, colorectal, kidney, lung, ovarian, pancreatic, or prostate cancer (Supplementary Fig. S1). Of the patients in the non-cancer (NC) group, 91 were asymptomatic (NCA) and the remaining 459 were patients presenting with generic symptoms (NCS), such as headache and stroke, as well as other benign conditions, e.g., non-malignant cysts and polyps. Patient serum samples were analysed using the Dxcover® Cancer Liquid Biopsy (Dxcover® Ltd., UK). Previously conducted studies have employed this technology, thus we direct the reader to these publications for further information [17, 19, 20].

### Algorithm training and cross-validation

Machine-learning models were developed to identify the cancerous signature from a known patient cohort and then predict the presence of cancer in an unknown population. A nested cross-validation (CV) strategy was used to develop the model to prevent data leakage and reduce sampling bias; the inner CV was used to tune the model hyperparameters, and the outer CV provided a robust test of model performance. In this approach, for the outer CV, patients were randomly split into training and test sets with a 70:30 split, repeated 51 times. Model hyperparameters were tuned to optimise the area under the receiver operating characteristic (ROC) curve and to give either a desired sensitivity or specificity, as estimated from fivefold CV on the training set (70%). The trained model was used to make predictions for the spectra in the test set (30%). Since each patient sample provides nine spectra, the final diagnosis was taken as the consensus prediction (maximum vote) from all nine spectra. Spectra from individual patients were not allowed to be present in both the training and test sets for a given resample. The classification metrics obtained from all 51 outer CV iterations were aggregated, and the mean and standard deviation of the resulting classification metrics were recorded.

### Metadata analysis

Breakdowns by patient metadata were performed as post hoc analyses using the test set predictions from all 51 train-test splits. The test set is randomly sampled from the full dataset, so a given patient would be expected to be present in approximately 15 test sets out of the 51 total. For each patient, the predictions from all test sets in which that patient is present were collected, and a detection rate was calculated as the ratio of correct predictions to the total number of predictions. The detection rates are then averaged over all patients of each category of metadata (e.g., disease stage).

### Primary analysis: organ-specific classifications

The individual cancer groups were tested against the full symptomatic non-cancer (NCS) dataset, where binary classifiers were built for each cancer type (Supplementary Table S2). Machine-learning models were developed to identify the cancerous signature from a known patient cohort and then predict the presence of cancer in an unknown population. The ovarian cancer set was compared against the female participants in the NCS group (NCS-F), and the prostate cancer group was examined against the male-only NCS patients (NCS-M), as described in Supplementary Table S3.

**Fig. 2:**
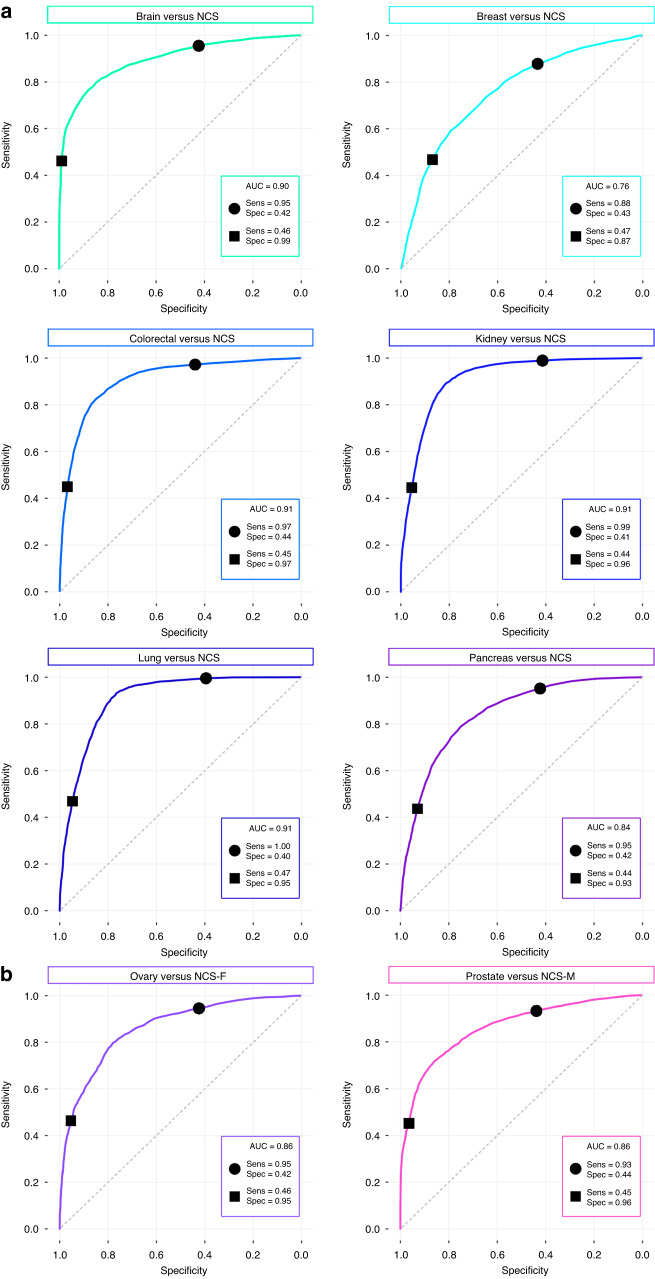
Mean receiver operating characteristic (ROC) curves for the organ-specific cancer classifications. In all ROC plots, the markers represent the sensitivity-tuned model (●), and specificity-tuned model (■), and AUC denotes the area under the curve. **a** Brain, breast, colorectal, kidney, lung, and pancreatic cancer versus non-cancer symptomatic (NCS), and **b** ovarian cancer versus NCS female-only (NCS-F) and prostate cancer versus NCS male-only (NCS-M).

Our technology enables us to tune the algorithm to achieve any value of sensitivity or specificity dictated by the receiver operating characteristic (ROC) curve. Probability thresholds were selected to achieve a minimum of 90% sensitivity or specificity for the (fivefold) CV of each cancer-specific classifier, and the sensitivity and specificity values for the resampled test sets have been reported (Supplementary Table S5). Subsequently, we tuned for greater sensitivity or specificity by targeting a minimum of 45% for the CV result, which was selected based on current commercially available triage-based liquid biopsies [21, 22]. The mean ROC curves for each of the organ-specific classifiers have been computed for patient predictions over 51 resampled test sets, e.g., 51 iterations of randomly selected train/test splits (Fig. 2).

### Pooled cancer classification

For our exploratory analysis of classifying ‘any cancer’ against non-cancer patients, samples were pooled for the training of two classification models: cancer versus all non-cancer (symptomatic and asymptomatic; C versus NC) and cancer versus non-cancer asymptomatic only (C versus NCA). ROC curves were generated, and the reported classification metrics include AUC, sensitivity and specificity. Classification metrics were then stratified by cancer stage for each model.

## Results

### Organ-specific classifications

For the primary analysis, the 90% CV sensitivity-tuned classifiers for lung (93% sensitivity/78% specificity) and kidney (92% sensitivity/79% specificity) cancer show real promise for cancer-specific applications with well-balanced statistics (Supplementary Table S5). For the specificity-tuned approach, the test strategy performed well for brain cancer (74% sensitivity/91% specificity), and colorectal cancer (77% sensitivity/90% specificity).

We targeted a minimum of 45% for the CV metrics, when maximising either sensitivity or specificity. The mean ROC curves are displayed in Fig. 2, showing the sensitivities and specificities for the sensitivity-tuned (●) specificity-tuned (■) models in each classifier. The brain, colorectal, kidney, and lung cancer versus NCS classifications reported very promising results, with area under the curve (AUC) of 0.90 and above. Pancreatic cancer versus NCS model achieved an AUC of 0.84. The breast cancer versus NCS model achieved an AUC of 0.76, which yields a sensitivity of 88% when specificity is 43%, and a specificity of 87% when sensitivity is 47%. The ovarian and prostate cancer models performed well, and both reported an AUC of 0.86. For each of the organ-specific classifications, the predictions were examined by cancer stage. The detection rates were calculated for both the sensitivity-tuned (Supplementary Table S6) and specificity-tuned (Supplementary Table S7) models, and the results are further discussed in the Supplementary Text section. As an exploratory analysis, indicative positive predictive values (PPV) for each cancer type have been determined for both the sensitivity-tuned and specificity-tuned models (Supplementary Table S8). PPVs ranging from 3.1 to 46.5% may be achievable if applied in an organ-specific screening programme for a population with a 2% prevalence of undetected cancer. On the other hand, based on symptomatic patients referred for cancer investigation in a hospital setting, with an estimated prevalence of 7% [7], PPVs between 10.5 and 75.1% may be observed depending on cancer type and diagnostic model selection. A reliable estimate of prevalence will be available after larger-scale prospective studies. For every classification in this study, 95% confidence intervals (CI) were calculated for each selected threshold on each ROC curve (Supplementary Table S9).

### Pooled cancer classification

**Fig. 3:**
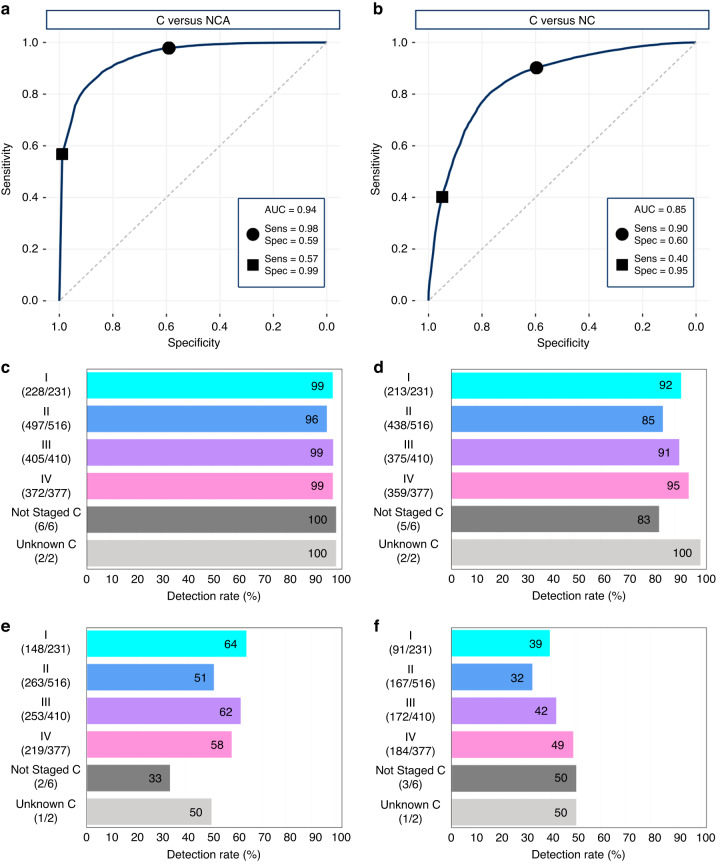
Results from the cancer (C) versus asymptomatic non-cancer (NCA) classification and the C versus all non-cancer (NC) classification. The mean receiver operating characteristic curve for **a** C versus NCA and **b** C versus NC showing the trade-off between sensitivity (Sens) and specificity (Spec), where the markers represent the sensitivity-tuned model (●), and specificity-tuned model (■), and AUC denotes the area under the curve. The detection rates for the sensitivity-tuned models **c**, **d** and the specificity-tuned models **e**, **f** are illustrated for the respective classifications, split by cancer stage.

The C versus NCA algorithm was tuned to selected thresholds that resulted in a 98% sensitivity or specificity for the CV set. The C versus NCA ROC analysis reported an AUC value of 0.94, which suggests excellent detection capability (Fig. 3a). This results in a 98% sensitivity (59% specificity) or a specificity of 99% (57% sensitivity). For the C versus NC dataset (Fig. 3b), the sensitivity-tuned model achieved 90% sensitivity and 60% specificity, and when tailored for greater specificity (95%) the sensitivity was 40%. The ROC curve generated an AUC of 0.85.

The bar graphs in Fig. 3 represent the detection rate when split by stage: (**c**), (**d**) sensitivity-tuned and (**e**), (**f**) specificity-tuned results for the C versus NCA and C versus NC classifiers, respectively. When exploring C versus NCA, the sensitivity-tuned model successfully predicted 98% of all cancers correctly. The detection rates were consistent across all stages: Stage I, 99%; II, 96%; III, 99%; IV, 99%. On the other hand, the high specificity (99%) model was still capable of detecting 64% of Stage I cancers and identified 51% of Stage II. Therefore, 55% of Stage I–II cancers were predicted correctly, highlighting the great potential for the Dxcover® Cancer Liquid Biopsy in the detection of early-stage cancers. The PPV for ‘all cancer’ was 4.3% for the sensitivity-tuned model. However, the specificity-tuned model may be better suited for a screening scenario. When screening for cancer in targeted populations a reasonable estimate of disease prevalence is around 2%, e.g., lung cancer screening programmes [23]. Therefore, with an assumed cancer prevalence of 2%, a PPV of 45% could be achieved with the specificity-tuned model.

For the overall C versus NC classification, Fig. 3d illustrates that when tuned for higher sensitivity, 92% (213/231) of Stage I and 85% (438/516) of Stage II cancers were detected. Similarly, the detection rate was extremely high for late-stage cancers—91% (375/410) and 95% (359/377) for Stage III and IV, respectively. For the model with 95% specificity, the detection rates are fairly consistent across Stages: I 39%; II 32%; III 42%; IV 49% (Fig. 3f). Patient metadata factors were explored to assess any impact on the predictions of the liquid biopsy. Patient age did not significantly affect either the sensitivity-tuned or specificity-tuned models; likewise, the detection rates for both models when split by sex did not indicate any concerns as a potential confounding factor (Supplementary Table S10).

### Feature importance

**Fig. 4:**
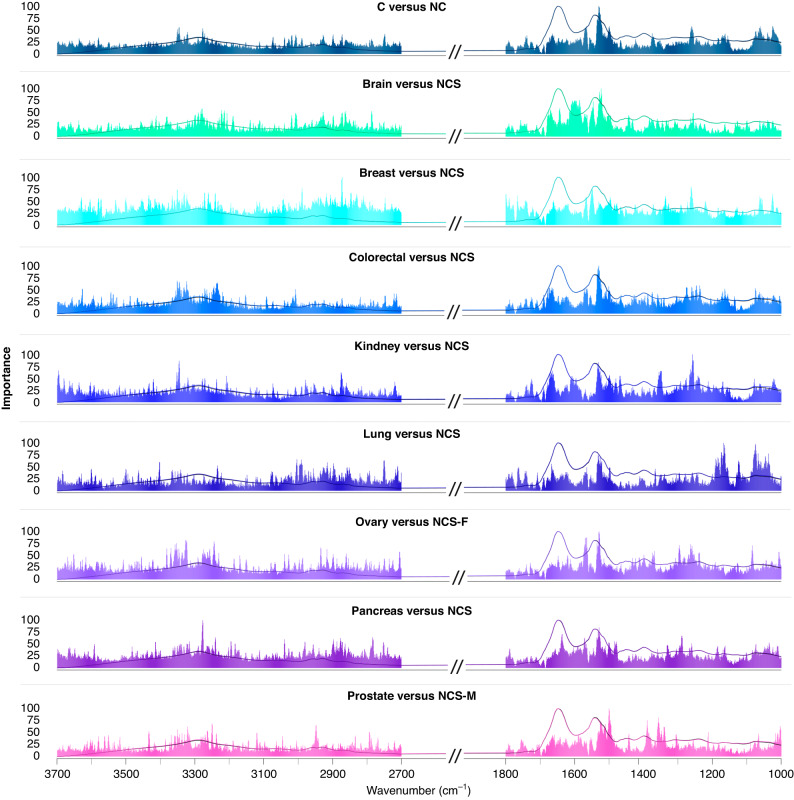
Feature importance plots highlighting the wavenumber regions that were found to be the most discriminatory for each organ-specific classification: non-cancer symptomatic (NCS), NCS female-only (NCS-F), NCS male-only (NCS-M). Note: the feature importance for cancer versus non-cancer has been included only for comparison.

When biological samples are irradiated with infrared (IR) light, stretching and bending of the bonds between chemical functional groups cause characteristic vibrations within these biomolecules [24]. A biological signature that represents the whole biochemical profile of that sample is generated, resulting in an IR spectrum. The spectral regions, or specific wavenumbers, that contribute to a classification can be assessed by feature importance analysis. The feature importance values were extracted from each classification, and Fig. 4 illustrates the wavenumber regions that were found to be the most discriminatory. The top five regions of importance are also described in Supplementary Table S11, with tentative biological assignments and their corresponding vibrational modes. The power of the technique lies in the use of the entire spectral signature, for which clear differences can be observed for the different cancers.

For C versus NC, the wavenumber region deemed to be of the highest importance was the Amide II (~1530 cm^−1^) band. This is one of the largest peaks in a serum spectrum, it contains information from overlapping bands associated with protein secondary structures, such as α-helices and β-sheets, thus variations in this region, as well as the Amide I region (1600–1700 cm^−1^), are often indicative of disease states [15]. The Amide II band is associated with N–H bending vibrations, and C–N stretching vibrations in protein molecules. In addition, N–H bending and C–N stretching vibrations (Amide III) and asymmetric P${{{{{{\rm{O}}}}}}}_{2}^{-}\,$ stretching in phosphodiesters were found to be important (~1260 cm^−1^). Other significant regions were ~1025 cm^−1^ (C–O and C–C stretching, C–OH deformation), ~1061 cm^−1^ (symmetric P${{{{{{\rm{O}}}}}}}_{2}^{-}\,$ stretching, C–O stretching), and ~3345 cm^−1^ (OH, C–H, N–H stretching). The brain cancer versus NCS classification reported similar importance within the Amide II and Amide A regions, as well as the Amide I band (~1607 cm^−1^, C = O and C–N stretching, N–H bending), but the lipid bands in the high wavenumber region were also shown to be significant in this model, which arise at ~2861 cm^−1^ and account for C–H and CH_2_ stretching vibrations. The top region for the breast cancer model was found around 2872 cm^−1^, accounting for C–OH deformation and C–O and C–C stretching vibrations which are related to glycogen and carbohydrates. The Amide II is deemed to be most important in the colorectal model, whereas the Amide III region (~1258 cm^−1^) was the highest importance for the kidney. Vibrations related to nucleic acids were also important for colorectal, and lipid vibrations were significant for the kidney cancer classifier. The lung cancer versus NCS feature importance seemed rather unique as most of the important bands appear in the lower end of the spectrum, mainly associated with symmetric (1074 cm^−1^) and asymmetric (1167 cm^−1^) P${{{{{{\rm{O}}}}}}}_{2}^{-}\,$ stretching vibrations, as well as lipidic C–H and CH_2_ stretching (2750 cm^−1^). Importance values for the ovarian and pancreatic cancer classifiers were also quite similar, as the top four wavenumbers regions are made up from proteinaceous regions. Lastly, the prostate versus NCS-M model were mainly associated with protein (Amide II/A) and lipid vibrations arising around ~1357 cm^−1^ and ~2947 cm^−1^.

## Discussion

The efficacy of the Dxcover® Cancer Liquid Biopsy heralds its potential to be employed as a rapid blood test for the earlier detection of various types of cancers. The spectroscopic liquid biopsy utilises an inclusive signal analysis which allows the interrogation of a wide range of molecules; such as lipids, carbohydrates, proteins, electrolytes and metabolites [15]. This generates a spectral signature that captures the full range of potential markers contained in human blood serum, combining both tumour- and immune-derived information. To our knowledge, this is the largest spectroscopic study to date, examining 8 different types of cancer. Our findings demonstrate the significant impact this low-cost test could have in supporting the earlier diagnosis of cancer.

Many leading technologies in the liquid biopsy field target high specificity at the expense of sensitivity [6], especially where a low-prevalence asymptomatic population is being screened. High specificity is required to reduce the number of false positive results that may trigger unnecessary investigations, but compromises sensitivity. Tumour-derived biomarkers, that are more abundant in late-stage cancer, such as cfDNA, are difficult to detect during the early-stages of disease, which is another potential reason for targeting specificity. A limitation of many studies is that the control sets tend to be comprised only of healthy individuals. By contrast, our organ-specific cancer classifiers were built using symptomatic non-cancer patients as the control group, meaning they are more representative of the patient cohort in whom the tests would likely be deployed. Despite this challenging cohort, high AUC values were reported for all cancers.

Single cancer detection tests are targeted at supporting rapid triage of symptomatic patients where the suspicion of cancer is low. However, not all cancers will require the same approach. The optimal test sensitivity and specificity for individual cancers may be influenced by the availability, costs and risks of subsequent diagnostic investigations, or whether the test would impact on a current screening programme [25]. The ‘tuneability’ of our approach will therefore be valuable for each individual cancer pathway. For example, the targets set by the Centers for Medicare & Medicaid Services for coverage of colorectal cancer tests is 74% sensitivity with 90% specificity [26], which we surpass in sensitivity (77%) when tuning the colorectal versus NCS model to 90% specificity. When discriminating between lung cancer and NCS patients, the performance of the sensitivity-tuned model is even comparable to other tests which use highly complex and expensive methodologies. For example, one study reported 94% sensitivity and 80% specificity when combing DNA testing with clinical risk factors, biomarker assays and computed tomography (CT) imaging [27]. Although, the results for the Dxcover® Cancer Liquid Biopsy are currently based solely on spectral data, in the future the diagnostic performance may be enhanced by combining our test with biomarkers and other clinical risk data. This requires further investigation.

Early cancer detection tests must effectively support the detection of early-stage tumours to be deemed valuable [28]. We have previously shown that this spectroscopic technology can detect tumours as small as 0.2 cm^3^, which indicates the test is sensitive to lesions that are small and also lower grade [29]. In this study, the Dxcover® Cancer Liquid Biopsy detected 64% of Stage I cancers (99% specificity) when targeting a patient cohort consisting of asymptomatic controls.

We have also demonstrated the ability to combat potential confounding factors with the overall C versus NC classification, by introducing symptomatic non-cancer patients. Various medical conditions are encompassed in the NCS set, such as inflammation and benign disease (as shown in Supplementary Data), yet the results were still encouraging. When specificity was 95%, the detection rates were Stages: I 39%; II 32%; III 42%; IV 49%. In a symptomatic patient population, it is likely high sensitivity would be preferred to enable a ‘rule-out’ test. The sensitivity-tuned approach enabled the detection of 92% (213/231) of Stage I and 85% (438/516) of Stage II cancers. An exploratory analysis to estimate the PPVs in a hospital setting reported values ranging between 3% and 71%, depending on the type of cancer and whether either sensitivity or specificity was maximised. As this is an early-stage study, it is difficult to accurately predict the disease prevalence, as details of how the test would be implemented in practice, that would determine the population of interest, and hence prevalence, have yet to be established.

The results presented here highlight the potential for this technology to be utilised as a liquid biopsy, yet it is important to note some limitations of the study. The spectroscopic method does not provide tumour genetic information in order to guide treatment. However, this can be easily mitigated though by employing a DNA-based test in patients subsequently demonstrated on imaging to have a cancer diagnosis. This would be more cost-effective than utilising the more expensive DNA-based test in unselected symptomatic patients. Another limitation of our spectroscopy test is that although we endeavoured to include control patients who were symptomatic with a wide range of benign and non-cancerous conditions, in this study all cancer types were classified against the same NCS group. Cancer-specific symptomatic control sets would be preferred, such as non-cancer patients presenting with abdominal pain and/or jaundice who are found to have pancreatitis or inflammation would be ideal comparators for pancreatic cancer. Likewise, patients with benign prostatic hyperplasia would be better suited for prostate cancer controls, or those with bowel polyps for colorectal cancer. A prospective collection (of cancer and cancer-specific controls) would allow the most accurate measures of sensitivity and specificity and examine any potential issues of comorbidities to be established. The patient cohort in this study was assembled with the intention of having 200 samples for each class, which has previously been reported as sufficient for reliable precision [30]. However, the test results would undoubtedly become more robust with a greater number of patients in each group.

The Dxcover® Cancer Liquid Biopsy is a simple, rapid blood test which could fit seamlessly into current diagnostic pathways. Blood serum testing already exists in medical laboratories for a variety of other diagnostic methods, thus the implementation of this test would not significantly disrupt clinical practice. Combining this pan-omic spectroscopic liquid biopsy with other orthogonal tests could provide an effective route to diagnosis, that is capable of detecting early-stage tumours. This could permit efficient triage of symptomatic patients, expediting further assessment for those most at-risk whilst excluding a cancer diagnosis in others. A proposed scenario of a combination approach is further explored in the Supplementary Text. Future studies are still required to validate the technology, which will comprise a greater number of patient samples, along with examining the potential of combinatorial pathways. Moreover, there may be potential to explore the use of the different spectral profiles for each cancer type for predicting tumour of origin, which will be assessed after large-scale trials. Studies are currently being planned with prospective patient recruitment and blinded analysis to truly assess the efficacy of the technique [14]. With further development, the Dxcover blood test could have a significant impact on the earlier detection of cancer.

## Conclusions

This discovery-stage study has demonstrated that the Dxcover® Cancer Liquid Biopsy platform has the potential to be deployed to detect multiple different types of cancer and, importantly, showed high sensitivity to Stage I and II disease, paving the way for earlier detection. There is a low barrier to integrating the blood test into existing diagnostic pathways since the technology is simple to use, minute sample volumes are required, and results can be provided to the requesting clinician rapidly. Combining the Dxcover® Cancer Liquid Biopsy with other diagnostic techniques—e.g., next-generation sequencing testing—may facilitate a cancer detection pathway that can offer superior sensitivity and specificity and be economically viable while significantly improving early-stage detection.

### Supplementary information

Supplementary Information File

Supplementary Data File

## Data Availability

The dataset analysed within the scope of the study cannot be published publicly due to privacy regulations under the General Data Protection Regulation (EU) 2016/679. The raw data includes clinical data from patients, clinical notes and information that could potentially compromise subjects’ privacy or consent. Non-confidential information may be available upon reasonable request.
