# Supplementary material for: A spectroscopic liquid biopsy for the earlier detection of multiple cancer types
Source: Br J Cancer. 2023 Sep 16;129(10):1658–66. doi: 10.1038/s41416-023-02423-7 (PMC10645969; doi:10.1038/s41416-023-02423-7)
Supplement: Supplementary file 1 — Supplementary Information File [file 41416_2023_2423_MOESM1_ESM.docx]

Supplementary Materials for

A spectroscopic liquid biopsy for the earlier detection of multiple cancer types

**Authors:** James M. Cameron^1†^, Alexandra Sala^1,2†^, Georgios Antoniou^1^, Paul M. Brennan^3^, Holly J. Butler^1^, Justin J.A. Conn^1^, Siobhan Connal^1,2^, Tom Curran^4^, Mark G. Hegarty^1^, Rose G. McHardy^1,2^, Daniel Orringer^5^, David S. Palmer^1,2^, Benjamin R. Smith^1^, Matthew J. Baker^1,6^*

† These authors contributed equally.

**Affiliations:**

^1^Dxcover Ltd., Royal College Building, 204 George Street, Glasgow, G1 1XW, UK

^2^Department of Pure and Applied Chemistry, Thomas Graham Building, 295 Cathedral Street, University of Strathclyde, Glasgow G11XL, UK

^3^Translational Neurosurgery, Centre for Clinical Brain Sciences, University of Edinburgh, Edinburgh, EH4 2XU, UK

^4^Children’s Mercy Research Institute at the Children’s Mercy Hospital, Kansas City, KS, USA

^5^Department of Neurosurgery, New York University Grossman School of Medicine, New York, NY 10018, USA

^6^School of Medicine, Faculty of Clinical and Biomedical Sciences, University of Central Lancashire, Preston, PR1 2HE, UK

*Corresponding author. Address: Suite RC534, Royal College Building, 204 George Street, Glasgow, G1 1XW. Tel: (+44)0141 548 4700. Email: [matthew.baker@dxcover.com](mailto:matthew.baker@dxcover.com)

**This PDF file includes:**

Methods

Supplementary Text

Supplementary References

Fig. S1

Tables S1 to S10

Supplementary Text

Supplementary Methods

*Patient Samples*

All samples sourced from patients eligible for inclusion in this study were sourced from biobanks. Cancer samples were gathered from the Wellcome Trust Clinical Research Facility at the Western General Hospital, Edinburgh, the Emergency Medicine Research Group (EMERGE) at the Edinburgh Royal Infirmary, The Beatson West of Scotland Cancer Centre in Glasgow, the University of Swansea, Royal Preston Hospital, and Manchester Cancer Research Centre. The study was designed to include approximately 200 serum samples for each individual class – which is sufficient for reliable precision[1] – and the stage distribution was selected to closely match the incidence and prevalence of tumors in the UK population [2]. All cancer samples were collected from patients with a confirmed cancer diagnosis according to the data collection methods of specified biobanks. Samples were collected before surgical resection or the start of other anti-cancer therapies. The non-cancer group was comprised of both asymptomatic controls and patients with suspicious symptomology. The primary analysis was planned to be agnostic as to whether control subjects were symptomatic or asymptomatic. It is intended to expand this research in future to allow investigation of potential differences in test performance between these groups. Blood samples were obtained with venipuncture using serum collection tubes; S-Monovette Z Gel (Sarstedt, Germany) and Vacutainer SST/SST II (BD, USA), and anonymized. Serum was extracted via centrifugation and stored in a -80°C freezer. Non-identifiable clinical and demographic data were obtained in-line with each biobank’s data control procedures.

*Patient Sample Analysis*

In this study, the serum samples sourced were stored at -80 °C until the date of analysis; samples were allowed to thaw for up to 30 minutes at room temperature (18-25 ºC) and inverted three times to ensure mixing and thawing before use. Each patient sample was prepared for analysis by pipetting 3 μL of serum onto each of the three sample wells of the Dxcover® Sample Slide (Dxcover® Ltd., UK). Prepared slides were placed in a drying unit incubator (Thermo Scientific™ Heratherm™, USA) at 35 °C for 1 hour, to control the dehydration process of the serum droplets[3]. Each dried sample slide was then inserted into the Dxcover® Autosampler (Dxcover® Ltd., UK) to be prepared for spectral collection. In this study, a PerkinElmer® Spectrum Two™ FTIR spectrometer (PerkinElmer® Inc., USA) was used to generate the spectral data (16 co-added scans at 4 cm^-1^ resolution with 1 cm^-1^ data spacing). A total of three spectra were collected for each sample well, resulting in nine replicates per patient, then submitted to the diagnostic algorithm to generate the disease prediction. Patient samples were reported as cancer positive or negative according to the diagnostic algorithm results.

Supplementary Results

*Organ-specific Classifications*

The sensitivity-tuned results are described in **Table S6**. The brain cancer detection rate was 100% (8/8) for grade I, 85% (23/27) for grade II, 86% (12/14) for grade III and 99% (191/192) for grade IV, which had the overwhelming majority for the brain cancer set. The breast cancer group had many more early-stage samples, and the detection rate was 96% (24/25) for stage I, 87% (79/91) for stage II, 89% (67/75) for stage III and 100% (9/9) for stage IV. The classifiers for colorectal, kidney and lung cancer all reported extremely high detection rates, between 98-100% for stage I and II. Despite being the smallest subset in this study, the ovarian cancer predictions were still highly promising: stage I 97% (30/31); II 86% (12/14); III 92% (47/51); IV 100% (29/29). Likewise, due to difficulty sourcing stage I pancreatic cancer samples there were only 8 included in the dataset, yet the sensitivity-tuned model was still capable of successfully predicting 7 of the stage I tumors (88%). Additionally, the detection rates for the other pancreatic cancer stages were 94% (II, 61/65) 99% (III, 71/72) and 95% (IV, 19/20). Lastly, the prostate cancer results further highlighted the potential of earlier detection: stage I 100% (4/4); II 93% (149/160); III 97% (30/31); IV 75% (3/4).

For the models that were tailored for a greater specificity (**Table S7**), more of the cancer samples were not detected. The brain cancer detection rates were low for grade I-III yet reported 52% (100/192) for grade IV (at 99% specificity). The breast cancer group had detection rates of 32% (8/25) for stage I, 47% (43/91) for stage II, 59% (44/75) for stage III, and 78% (7/9) for stage IV. The colorectal classifier reported the highest stage I detection rate for the specificity-tuned models with 18 out of 36 being predicted correctly (50%). Furthermore, the colorectal stage II, III and IV detection rates were 36% (25/70), 51% (34/67) and 44% (12/27), respectively. Kidney cancer reported the highest detection rate for stage II (66%, 19/29), and the remaining stages were: stage I 46% (40/87); III 38% (13/34); IV 29% (15/51). The detection rates for lung cancer were 35% (11/31) for stage I, 40% (24/60) for stage II, 60% (39/65) for stage III and 44% (20/45) for stage IV. The detection rates were similar for ovarian cancer – stage I 39% (12/31), II 36% (5/14), III 53% (27/51), IV 45% (13/29) – and pancreatic cancer – stage I 38% (3/8), II 37% (24/65), III 44% (32/72), IV 55% (11/20). Finally, the prostate cancer performance was respectable for stage II (43%, 68/160) and III (58%, 18/31), but low for stage I and IV which is likely to be attributed to the very small number of samples in those groups (n = 4).

Supplementary Discussion

*Combination Scenario*

Using the sensitivity-tuned model as a first-line cancer detection test could capture the majority of cancer patients and fast-track patients with a positive result into further testing (e.g., genetic-based analysis) whilst quickly ruling out 60% of the patients without disease. In theory, this could provide a focused, enriched cohort for a second-line test and due to the minute volume used (only 9 μL required), the additional testing could use the same patient blood draw. The impact on patients of a combined testing regime on a single blood draw can be estimated. Based on a 100,000 patient population with a 2% disease prevalence (projected prevalence in the USA in 2040)[4]. 2,000 patients would have cancer and the remaining 98,000 would not. Implementing a high sensitivity first-line cancer detection test (90% sensitivity / 60% specificity) would mean that 41,000 patients will test positive and proceed to a second-line test, such as next generation sequencing (NGS). At this stage, the disease prevalence would be 4.4%, more than twice than the original population. The other 59,000 patients would test negative and not progress to second-line testing, meaning they can be ruled out in a much shorter time (1-2 days compared to 10+ days), with disease prevalence in the negative cohort of 0.3% which is six times less than in the original population.

**Supplementary References**

1. Beleites C, Neugebauer U, Bocklitz T, Krafft C, Popp J. Sample size planning for classification models. Analytica Chimica Acta. 2013;760:25–33.

2. Cancer Research UK. Types of Cancer [Internet]. Accessed August 2022. Available from: http://www.cancerresearchuk.org/about-cancer/what-is-cancer/how-cancer-starts/types-of-cancer

3. Cameron JM, Butler HJ, Palmer DS, Baker MJ. Biofluid spectroscopic disease diagnostics: A review on the processes and spectral impact of drying. Journal of Biophotonics. 2018;11:e201700299.

4. GLOBOCAN. Globocan Cancer Tomorrow [Internet]. Accessed November 2021. Available from: https://gco.iarc.fr/tomorrow/en/dataviz/tables?populations=900_840&age_start=10

Supplementary Figures


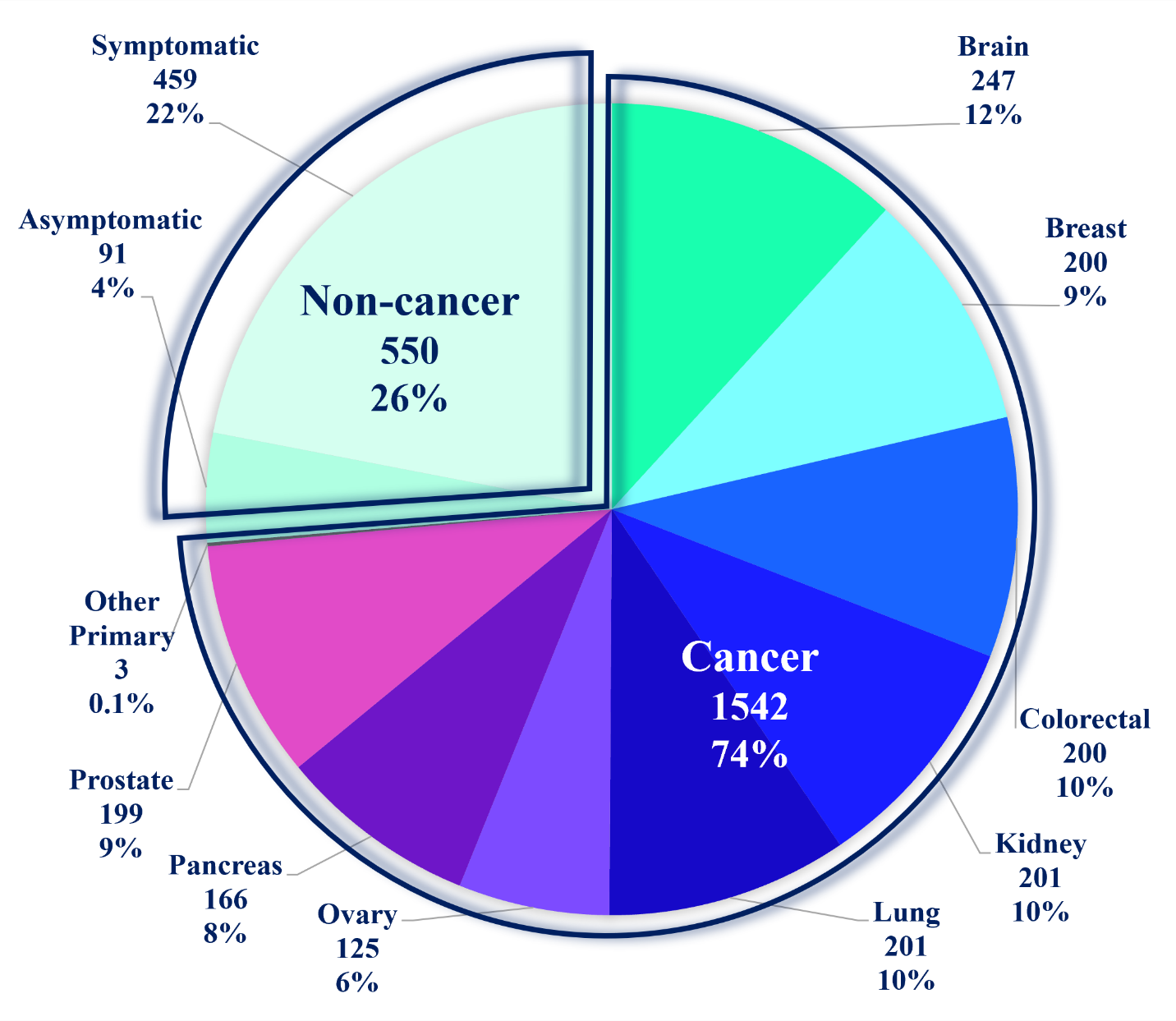


**Fig. S1.** Schematic breakdown of the full patient cohort. Cancer set is comprised of patients with brain, breast, colorectal, kidney, lung, ovarian, pancreatic, and prostate cancer. Non-cancer group includes asymptomatic participants as well as symptomatic patients with non-malignant disease.

Supplementary Tables

Table S1. Patient demographics for the full patient cohort.

|  | **Cancer (C)** | **Asymptomatic  Non-cancer (NCA)** | **Non-cancer (NC)** | **Total**^†^ |
| --- | --- | --- | --- | --- |
|  | *(n = 1542)* | *(n = 91)* | *(n = 550)* | *(n = 2092)* |
| **Age, years** |  |  |  |  |
| Mean | 63 | 41 | 54 | 61 |
| Min – max | 20 – 90 | 20 – 82 | 20 – 90 | 20 – 90 |
| **Sex, n (%)** |  |  |  |  |
| Female | 748 (49) | 50 (55) | 310 (56) | 1058 (51) |
| Male | 794 (51) | 41 (45) | 240 (44) | 1034 (49) |
| **Cancer stage, n (%)** |  |  |  |  |
| I | 231 (15) | - | - | 231 (11) |
| II | 516 (33) | - | - | 516 (25) |
| III | 410 (27) | - | - | 410 (20) |
| IV | 377 (24) | - | - | 377 (18) |
| Not staged* | 6 (1) | 91 (100) | 550 (100) | 556 (26) |
| Unknown** | 2 (0) | - | - | 2 (0) |

^†^Total includes C and NC only; NCA patients are comprised within the NC set.

* This group does not require staging (or grading).

** Cancer stage and/or staging information has not been recorded.

Table S2.

Patient demographics for the organ specific cancer v non-cancer symptomatic (NCS) classifications.

|  | **Brain†** | **Breast** | **Colorectal** | **Kidney** | **Lung** | **Pancreas** | **NCS** |
| --- | --- | --- | --- | --- | --- | --- | --- |
|  | *(n = 247)* | *(n = 200)* | *(n = 200)* | *(n = 201)* | *(n = 201)* | *(n = 166)* | *(n = 459)* |
| **Age, years** |  |  |  |  |  |  |  |
| Mean | 60 | 62 | 66 | 62 | 66 | 65 | 57 |
| Min - max | 22 -84 | 28 - 90 | 29 - 85 | 25 - 87 | 20 - 86 | 39 - 87 | 20 - 90 |
| **Sex, n (%)** |  |  |  |  |  |  |  |
| Female | 123 (50) | 196 (98) | 64 (32) | 75 (37) | 95 (47) | 69 (42) | 260 (57) |
| Male | 124 (50) | 4 (2) | 136 (68) | 126 (63) | 106 (53) | 97 (58) | 199 (43) |
| **Cancer stage, n (%)** |  |  |  |  |  |  |  |
| I | 8 (3) | 25 (13) | 36 (18) | 87 (43) | 31 (16) | 8 (5) | - |
| II | 27 (11) | 91 (45) | 70 (35) | 29 (15) | 60 (30) | 65 (39) | - |
| III | 14 (6) | 75 (37) | 67 (33) | 34 (17) | 65 (32) | 72 (43) | - |
| IV | 192 (78) | 9 (5) | 27 (14) | 51 (25) | 45 (22) | 20 (12) | - |
| Not staged* | 6 (2) | - | - | - | - | - | 459 (100) |
| Unknown** | - | - | - | - | - | 1 (1) | - |

**†** Brain cancers split by grade, as described in Table S4.

* This group does not require staging (or grading).

** Cancer stage and/or staging information has not been recorded.

Table S3.

Patient demographics for the organ specific cancer classifications for ovarian and prostate cancer against the sex-specific non-cancer symptomatic (NCS) groups.

|  | **Ovary** | **Prostate** | **NCS Females Only (NCS-F)** | **NCS Males Only (NCS-M)** |
| --- | --- | --- | --- | --- |
|  | *(n = 125)* | *(n = 199)* | *(n = 260)* | *(n = 199)* |
| **Age, years** |  |  |  |  |
| Mean | 61 | 62 | 56 | 59 |
| Min - max | 21 - 88 | 43 - 87 | 20 - 90 | 20 - 90 |
| **Sex, n (%)** |  |  |  |  |
| Female | 125 (100) | - | 260 (100) | - |
| Male | - | 199 (100) | - | 199 (100) |
| **Cancer stage, n (%)** |  |  |  |  |
| I | 31 (25) | 4 (2) | - | - |
| II | 14 (11) | 160 (80) | - | - |
| III | 51 (41) | 31 (16) | - | - |
| IV | 29 (23) | 4 (2) | - | - |
| Not staged* | - | - | 260 (100) | 199 (100) |

* This group does not require staging.

Table S4.
Summary of brain cancer types split by tumor grade.

| **WHO Grade** | **Brain Tumor Type** | **Number of patients** |
| --- | --- | --- |
| I | Meningioma | 6 |
|  | Schwannoma | 1 |
|  | Subependymoma | 1 |
| II | Astrocytoma | 8 |
|  | Ependymoma | 1 |
|  | Meningioma | 10 |
|  | Oligodendroglioma | 7 |
|  | Pituitary Adenoma | 1 |
| III | Astrocytoma | 7 |
|  | Medulloblastoma | 1 |
|  | Oligodendroglioma | 6 |
| IV | Glioblastoma | 148 |
|  | Gliosarcoma | 2 |
|  | Medulloblastoma | 2 |
|  | Metastatic | 40 |

Table S5.

Sensitivity and specificity values for the resampled test sets for each of the organ-specific classifications. The results here are based upon thresholds chosen where either sensitivity or specificity was a minimum value of 90% for the cross-validation. Non-cancer symptomatic (NCS), NCS female-only (NCS-F) and NCS male-only (NCS-M).

| Organ Specific Classifier | Sensitivity-tuned | | Specificity-tuned | |
| --- | --- | --- | --- | --- |
|  | Sensitivity (%) | Specificity (%) | Sensitivity (%) | Specificity (%) |
| Brain v NCS | 91 | 64 | 74 | 91 |
| Breast v NCS | 93 | 31 | 38 | 93 |
| Colorectal v NCS | 91 | 76 | 77 | 90 |
| Kidney v NCS | 92 | 79 | 71 | 91 |
| Lung v NCS | 93 | 78 | 69 | 90 |
| Ovary v NCS-F | 92 | 54 | 58 | 90 |
| Pancreas v NCS | 92 | 52 | 57 | 90 |
| Prostate v NCS-M | 92 | 51 | 66 | 93 |

**Table S6**. Detection rates from the organ-specific classifications for the sensitivity-tuned models, broken down by stage, based upon the models with a lower limit of 45% specificity for the cross validation.

| **Cancer Type** | **Stage** | **Actual** | **Identified Correctly** | **Detection Rate (%)** |
| --- | --- | --- | --- | --- |
| Brain**^†^** | I | 8 | 8 | 100 |
|  | II | 27 | 23 | 85 |
|  | III | 14 | 12 | 86 |
|  | IV | 192 | 191 | 99 |
|  | Not Graded ^*^ | 6 | 5 | 83 |
| Breast | I | 25 | 24 | 96 |
|  | II | 91 | 79 | 87 |
|  | III | 75 | 67 | 89 |
|  | IV | 9 | 9 | 100 |
| Colorectal | I | 36 | 36 | 100 |
|  | II | 70 | 70 | 100 |
|  | III | 67 | 65 | 97 |
|  | IV | 27 | 24 | 89 |
| Kidney | I | 87 | 86 | 99 |
|  | II | 29 | 29 | 100 |
|  | III | 34 | 34 | 100 |
|  | IV | 51 | 50 | 98 |
| Lung | I | 31 | 31 | 100 |
|  | II | 60 | 59 | 98 |
|  | III | 65 | 65 | 100 |
|  | IV | 45 | 45 | 100 |
| Ovary | I | 31 | 30 | 97 |
|  | II | 14 | 12 | 86 |
|  | III | 51 | 47 | 92 |
|  | IV | 29 | 29 | 100 |
| Pancreas | I | 8 | 7 | 88 |
|  | II | 65 | 61 | 94 |
|  | III | 72 | 71 | 99 |
|  | IV | 20 | 19 | 95 |
|  | Unknown^**^ | 1 | 1 | 100 |
| Prostate | I | 4 | 4 | 100 |
|  | II | 160 | 149 | 93 |
|  | III | 31 | 30 | 97 |
|  | IV | 4 | 3 | 75 |

**†** Brain cancers split by grade, as described in Table S4.

* This group of cancers do not require grading.

** Cancer stage and/or staging information has not been recorded.

Table S7.

Detection rates from the organ-specific classifications, for the specificity-tuned models split by stage. The results here are based upon a lower limit of 45% sensitivity for the cross-validation.

| **Cancer Type** | **Stage** | **Actual** | **Identified Correctly** | **Detection Rate (%)** |
| --- | --- | --- | --- | --- |
| Brain**^†^** | I | 8 | 2 | 25 |
|  | II | 27 | 4 | 15 |
|  | III | 14 | 3 | 21 |
|  | IV | 192 | 100 | 52 |
|  | Not Graded ^*^ | 6 | 2 | 33 |
| Breast | I | 25 | 8 | 32 |
|  | II | 91 | 43 | 47 |
|  | III | 75 | 44 | 59 |
|  | IV | 9 | 7 | 78 |
| Colorectal | I | 36 | 18 | 50 |
|  | II | 70 | 25 | 36 |
|  | III | 67 | 34 | 51 |
|  | IV | 27 | 12 | 44 |
| Kidney | I | 87 | 40 | 46 |
|  | II | 29 | 19 | 66 |
|  | III | 34 | 13 | 38 |
|  | IV | 51 | 15 | 29 |
| Lung | I | 31 | 11 | 35 |
|  | II | 60 | 24 | 40 |
|  | III | 65 | 39 | 60 |
|  | IV | 45 | 20 | 44 |
| Ovary | I | 31 | 12 | 39 |
|  | II | 14 | 5 | 36 |
|  | III | 51 | 27 | 53 |
|  | IV | 29 | 13 | 45 |
| Pancreas | I | 8 | 3 | 38 |
|  | II | 65 | 24 | 37 |
|  | III | 72 | 32 | 44 |
|  | IV | 20 | 11 | 55 |
|  | Unknown^**^ | 1 | 1 | 100 |
| Prostate | I | 4 | 1 | 25 |
|  | II | 160 | 68 | 43 |
|  | III | 31 | 18 | 58 |
|  | IV | 4 | 1 | 25 |

**†** Brain cancers split by grade, as described in Table S4.

* This group of cancers do not require grading.

** Cancer stage and/or staging information has not been recorded.

**Table S8.** Positive predictive values (PPV) split by cancer type, for sensitivity-tuned and specificity-tuned models. PPVs have been calculated by assuming 2% disease prevalence for a screening population and 7% prevalence for a symptomatic setting.

| Cancer Type | Screening scenario (est. 2% prevalence) | | Symptomatic scenario (est. 7% prevalence) | |
| --- | --- | --- | --- | --- |
|  | PPV  (Sensitivity-tuned) | PPV  (Specificity-tuned) | PPV  (Sensitivity-tuned) | PPV  (Specificity-tuned) |
| Brain | 3.3% | 46.5% | 11.2% | 75.1% |
| Breast | 3.1% | 7.6% | 10.5% | 23.2% |
| Colorectal | 3.4% | 23.7% | 11.6% | 53.3% |
| Kidney | 3.3% | 19.6% | 11.2% | 47.0% |
| Lung | 3.3% | 15.6% | 11.0% | 40.2% |
| Ovary | 3.1% | 12.5% | 10.7% | 34.4% |
| Pancreas | 3.2% | 15.8% | 10.9% | 40.8% |
| Prostate | 3.3% | 19.2% | 11.2% | 46.5% |

**Table S9.** 95% confidence intervals (CI) for a one-sample Student's t-test, carried out for each selected threshold on the presented receiver operating characteristic curves for every classification.

| Model | Tuning | CI Sens (%) | CI Spec (%) |
| --- | --- | --- | --- |
|  |  |  |  |
| C v NCA | 98% Sens | (97.6, 98.0) | (56.9, 61.2) |
|  | 98% Spec | (55.7, 57.8) | (98.1, 99.7) |
| C v NC | 90% Sens | (89.8, 90.6) | (58.8, 60.5) |
|  | 95% Spec | (39.3, 40.9) | (94.3, 95.3) |
| Brain v NCS | 45% Sens | (44.6, 47.6) | (98.8, 99.3) |
|  | 45% Spec | (94.7, 96.3) | (40.8, 43.9) |
|  | 90% Sens | (89.0, 90.8) | (61.0, 63.8) |
|  | 90% Spec | (72.4, 75.2) | (89.6, 90.7) |
| Breast v NCS | 45% Sens | (44.9, 48.7) | (85.9, 87.8) |
|  | 45% Spec | (86.8, 88.9) | (42.5, 44.5) |
|  | 90% Sens | (91.7, 93.6) | (30.5, 32.6) |
|  | 90% Spec | (34.8, 37.5) | (90.8, 92.4) |
| Colorectal v NCS | 45% Sens | (43.3, 46.5) | (96.3, 97.2) |
|  | 45% Spec | (96.6, 97.8) | (42.4, 45.5) |
|  | 90% Sens | (89.5, 91.4) | (73.7, 75.8) |
|  | 90% Spec | (73.4, 76.7) | (88.9, 90.5) |
| Kidney v NCS | 45% Sens | (42.9, 46.0) | (95.0, 96.1) |
|  | 45% Spec | (98.6, 99.3) | (40.3, 42.5) |
|  | 90% Sens | (90.4, 92.3) | (77.7, 79.4) |
|  | 90% Spec | (68.7, 71.4) | (89.5, 90.9) |
| Lung v NCS | 45% Sens | (45.1, 48.6) | (94.1, 95.3) |
|  | 45% Spec | (99.3, 99.8) | (38.2, 40.9) |
|  | 90% Sens | (90.7, 92.8) | (76.7, 78.6) |
|  | 90% Spec | (65.7, 69.2) | (88.6, 90.1) |
| Ovary v NCS-F | 45% Sens | (44.3, 48.4) | (94.7, 96.1) |
|  | 45% Spec | (93.4, 95.6) | (41.0, 43.9) |
|  | 90% Sens | (90.8, 93.2) | (52.0, 55.3) |
|  | 90% Spec | (58.6, 62.3) | (88.5, 90.4) |
| Pancreas v NCS | 45% Sens | (41.6, 45.7) | (92.2, 93.7) |
|  | 45% Spec | (94.3, 96.0) | (40.9, 43.6) |
|  | 90% Sens | (91.0, 93.0) | (50.6, 53.3) |
|  | 90% Spec | (51.9, 55.9) | (89.0, 90.7) |
| Prostate v NCS-M | 45% Sens | (43.5, 46.7) | (95.8, 97.2) |
|  | 45% Spec | (92.3, 94.2) | (42.1, 45.6) |
|  | 90% Sens | (90.3, 92.5) | (49.1, 53.0) |
|  | 90% Spec | (63.2, 66.6) | (89.8, 91.8) |

Table S10.

Detection rates for the cancer (C) v non-cancer (NC) sensitivity and specificity-tuned models split by age and sex.

|  | | No. of Patients | | **Correctly identified *n* (%)** | | | |
| --- | --- | --- | --- | --- | --- | --- | --- |
|  |  |  |  | Sensitivity-tuned Model  (90% Sensitivity) | | Specificity-tuned Model (95% Specificity) | |
|  |  | C | NC | C | NC | C | NC |
| Sex | Male | 794 | 240 | 732 (92) | 153 (64) | 327 (41) | 229 (95) |
|  | Female | 748 | 310 | 660 (88) | 184 (59) | 291 (40) | 296 (95) |
| Age (deciles) | 20 | 15 | 52 | 13 (87) | 42 (81) | 6 (40) | 52 (100) |
|  | 30 | 37 | 65 | 28 (76) | 48 (74) | 14 (38) | 65 (100) |
|  | 40 | 140 | 66 | 118 (84) | 44 (67) | 47 (34) | 62 (94) |
|  | 50 | 331 | 84 | 294 (89) | 49 (58) | 118 (36) | 81 (96) |
|  | 60 | 539 | 105 | 490 (91) | 52 (50) | 219 (41) | 98 (93) |
|  | 70 | 373 | 116 | 347 (93) | 63 (54) | 170 (46) | 107 (92) |
|  | 80 | 104 | 54 | 100 (96) | 34 (63) | 43 (41) | 53 (98) |
|  | 90 | 3 | 4 | 2 (67) | 2 (50) | 1 (33) | 3 (75) |

**Table S11.**

The top 5 wavenumber regions which were found to be the most discriminatory for each of the binary classifications, with their corresponding tentative biological assignments and vibrational modes.

| **Classification** | **Approximate wavenumber (cm^-1^)** | **Importance** | **Biological assignments** | **Vibrational Modes** |
| --- | --- | --- | --- | --- |
| **C v NC** | 1530 | 100 | Amide II of Proteins | N-H bending, C-N stretching |
|  | 1260 | 58 | Amide III of Proteins, Phosphodiesters | N-H in-plane bend, C-N stretching, asymmetric P$O_{2}^{-}$stretching |
|  | 1025 | 58 | Glycogen, Carbohydrates | C-O and C-C stretching, C-OH deformation |
|  | 1061 | 57 | Nucleic acids, Phosphodiesters | Symmetric P$O_{2}^{-}$stretching, C-O stretching |
|  | 3345 | 56 | Amide A of Proteins | OH, C-H, N-H stretching |
| **Brain v NCS** | 1523 | 100 | Amide II of Proteins | N-H bending, C-N stretching |
|  | 1607 | 75 | Amide I of Proteins | C=O and C-N stretching, N-H bending |
|  | 3278 | 58 | Proteins (Amide A), Nucleic acids | Symmetric O-H stretching, N-H stretching |
|  | 2861 | 52 | Lipids | C-H, CH_2_ stretching |
|  | 1256 | 49 | Amide III of Proteins, Phosphodiesters | N-H in-plane bend, C-N stretching, asymmetric P$O_{2}^{-}$stretching |
| **Breast v NCS** | 2872 | 100 | Glycogen, Carbohydrates | C-O and C-C stretching, C-OH deformation |
|  | 1261 | 80 | Amide III of Proteins, Phosphodiesters | N-H in-plane bend, C-N stretching, asymmetric P$O_{2}^{-}$stretching |
|  | 1549 | 76 | Amide II of Proteins | N-H bending, C-N stretching |
|  | 3351 | 74 | Amide A of Proteins | OH, C-H, N-H stretching |
|  | 1025 | 65 | Glycogen, Carbohydrates | C-O and C-C stretching, C-OH deformation |
| **Colorectal v NCS** | 1530 | 100 | Amide II of Proteins | N-H bending, C-N stretching |
|  | 3351 | 69 | Amide A of Proteins | O-H, C-H, N-H stretching |
|  | 3234 | 65 | Proteins (Amide A), Nucleic acids | Symmetric O-H stretching, N-H stretching |
|  | 1246 | 59 | Amide III of Proteins, Phosphodiesters | N-H in-plane bend, C-N stretching, asymmetric P$O_{2}^{-}$stretching |
|  | 1666 | 54 | Amide I of Proteins | C=O and C-N stretching, N-H bending |
| **Kidney v NCS** | 1258 | 100 | Amide III of Proteins, Phosphodiesters | N-H in-plane bend, C-N stretching, asymmetric P$O_{2}^{-}$stretching |
|  | 3345 | 86 | Amide A of Proteins | O-H, C-H, N-H stretching |
|  | 1531 | 83 | Amide II of Proteins | N-H bending, C-N stretching |
|  | 1348 | 65 | Lipids, Proteins | C-O stretching, C-H and N-H deformation |
|  | 1664 | 65 | Amide I of Proteins | C=O and C-N stretching, N-H bending |
| **Lung v NCS** | 1167 | 100 | Nucleic Acids | Asymmetric P$O_{2}^{-}$stretching |
|  | 1074 | 98 | Nucleic acids, Phosphodiesters | Symmetric P$O_{2}^{-}$stretching, C-O stretching |
|  | 1532 | 74 | Amide II of Proteins | N-H bending, C-N stretching |
|  | 2750 | 66 | Lipids | C-H, CH_2_ stretching |
|  | 1124 | 63 | Carbohydrates | C-O and C-C stretching |
| **Ovary v NCS-F** | 1529 | 100 | Amide II of Proteins | N-H bending, C-N stretching |
|  | 3327 | 82 | Amide A of Proteins | O-H, C-H, N-H stretching |
|  | 3244 | 79 | Proteins (Amide A), Nucleic acids | Symmetric O-H stretching, N-H stretching |
|  | 1263 | 74 | Amide III of Proteins, Phosphodiesters | N-H in-plane bend, C-N stretching, asymmetric P$O_{2}^{-}$stretching |
|  | 1084 | 70 | Nucleic acids, Phosphodiesters | Symmetric P$O_{2}^{-}$stretching, C-O stretching |
| **Pancreas v NCS** | 3277 | 100 | Proteins (Amide A), Nucleic acids | Symmetric O-H stretching, N-H stretching |
|  | 1529 | 84 | Amide II of Proteins | N-H bending, C-N stretching |
|  | 1636 | 71 | Amide I of Proteins | C=O and C-N stretching, N-H bending |
|  | 1288 | 67 | Amide III of Proteins, Phosphodiesters | N-H in-plane bend, C-N stretching, asymmetric P$O_{2}^{-}$stretching |
|  | 2784 | 64 | Lipids | C-H, CH_2_ stretching |
